# A New Antirotation Strategy of K-Wire Tension Band Therapy for Patellar Fracture

**DOI:** 10.3389/fsurg.2022.891869

**Published:** 2022-05-10

**Authors:** Fengpo Sun, Yawen Zhang, Quan Ji, Tongyi Zhang, Yi Zhu, Ze Zhang, Ruining Han, Liangyuan Wen

**Affiliations:** Department of Orthopedics, Beijing Hospital, National Center of Gerontology, Institute of Geriatric Medicine, Chinese Academy of Medical Sciences, Beijing, China

**Keywords:** patellar fracture, tension band method, antirotation, internal fixation, trauma

## Abstract

**Background:**

Patellar fracture is a common phenomenon observed in orthopedic clinics. Many methods have been shown to be effective in the fixation of patellar fracture. However, there are few studies on the antirotation effect of these methods. The purpose of this study is to present a new strategy of K-wire tension band therapy for patellar fracture and explore the antirotation effect of the modified tension band method on patellar fracture.

**Methods:**

A retrospective clinical observation study was conducted on 75 patients with patellar fracture. Totally, 46 patients were enrolled to the traditional group, who received the traditional K-wire tension band therapy. The modified group included 29 patients on whom our new strategy was implemented. The operation time, intraoperative blood loss, and fracture healing time were collected to compare the two operations and the knee society score (KSS) scores after the operations, and complications were recorded and retrieved to indicate the effectiveness of the two treatments.

**Results:**

The preoperative baseline data (gender, age, fracture types) of the two groups showed no significant statistical difference. Similarly, there was no significant difference in the operation time, intraoperative blood loss, and fracture healing time between the two groups. The KSS clinical scores 1 year after operation was 90 (84, 95) for the traditional group as compared with 99 (97, 100) for the modified group (*p* < 0.05). The KSS functional scores 1 year after operation in the two groups were 90 (65, 90) and 100 (90, 100) (*p* < 0.05). The incidences of complications due to the rotation of K-wires in the traditional group and the modified group were 76.1% (35 of 46) and 6.9% (2 of 29) with a significant statistical difference (*p* < 0.05).

**Conclusion:**

This study shows that our modified tension band therapy is an effective strategy for antirotation in the treatment of patellar fracture and proves that it can achieve better clinical outcomes than the traditional K-wire tension band method. This new strategy may be a safe and effective clinical technique for the treatment of patellar fracture. However, more prospective randomized controlled trials with larger sample sizes are still needed to further prove its efficacy.

## Background

Patellar fractures are relatively common injuries in adults destroying the continuity of the extensor mechanism of the knee and accounting for about 1% of all skeletal fractures (1). The patellar is the largest sesamoid in the human body that can increase the lever arm of quadriceps femoris. This can add an additional 60% of the force needed to gain full extension (2). Operation is necessary for patellar fractures, especially for displaced or step-off fractures above 2 mm, in order to regain the full force of the knee extension. The most popular methods for patellar operation include the K-wire tension band, cable pin, and cannulated screws (3). The K-wire tension band method is extensively applied for all types of patellar fracture due to its excellent prognosis. Favorable outcomes in this method for patellar fracture have been reported (4, 5). But the incidence of complications such as skin prominence and ulceration due to the rotation of K-wires is rather high (6, 7). These complications usually affect rehabilitation efforts, necessitating a second operation to remove the hardware. So, we invented a new K-wire tension band method to avoid the rotation of K-wires and reduce the related incidence of complications.

To ascertain the effectiveness of this new technique in the treatment of patellar fracture, we conducted a retrospective cohort study to evaluate the postoperative outcome of patellar fracture patients using the modified K-wire tension band method and the traditional K-wire tension band method. The main purpose of our study was to compare the antirotation performance of the two methods.

## Materials and Method

### Patients

Between January 2016 and December 2018, 75 patients with patellar fracture were treated either with the traditional K-wire tension band method or with the modified method. The traditional K-wire tension band method was used in patients before October 2017, and patients from November 2017 to December 2018 underwent the modified method. The clinical data of the patients including gender, age, fracture type, operation time, intraoperative blood loss, fracture healing time, knee society score (KSS) scores after operation and complications were recorded and analyzed in our study. The complications due to the rotation of K-wires were recorded, including skin prominence or damage or the limitation of knee flexion movement. The primary outcome was the rotation of K-wires. The secondary outcome was the symptoms that occurred due to the rotation of the K-wires, including skin irritability and ulceration and fixation failure, including the breakage of the K-wires or titanium cables, and titanium cables unhooking the connection of the K-wires.

### Instruments

The 2.0-mm titanium K-wires and titanium cables used in this study were obtained from DePuy Synthes company. Studies have shown that the most useful of these materials is titanium. The use of titanium eliminates the possibility of allergic reactions to nickel in other materials (8).

### Procedure

We used this new strategy in compliance with the principles of the Declaration of China. The protocol of this study was reviewed and approved by the Institutional Review Board of Beijing Hospital (2018BJYYEC-032-05).

Under general or spinal anesthesia, surgery was performed in supine position. The tourniquet was used in each case. An incision was made along the longitudinal axis of the knee joint. After the patellar was presented, the fracture was then reduced with reduction forceps. Two 2.0-mm K-wires were inserted into the fragments to fix the fracture. The titanium cable was then placed as a figure-of-eight pattern around the K-wires in front of the patellar. More K-wires were used according to the fracture type. According to the type of group by which the patients were divided, we used the traditional K-wire band method or the modified method to fix the fracture.

**Figure 1:**
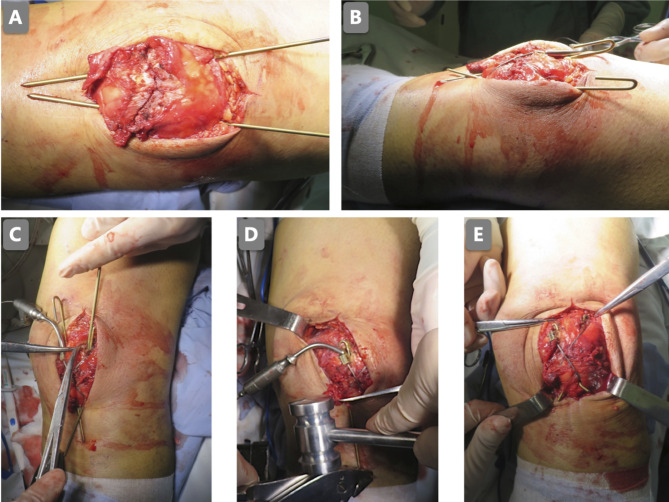
The patellar was fixed with two titanium K-wires with a titanium cable (**A**); the proximal K-wires were bent to 180° (**B**); the holes in the superior extreme of the patellar were drilled next to the bent part of the K-wires (**C**); the K-wires were knocked into the holes (**D**); the 1-mm titanium cable was placed as a figure-of-eight pattern (**E**).

**Figure 2:**
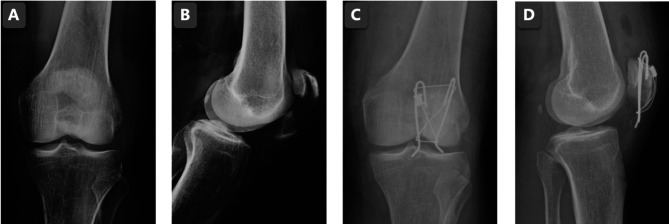
The AP knee joint X-ray before the surgery (**A**); the lateral knee joint X-ray before the surgery (**B**); the AP knee joint X-ray post the modified method surgery (**C**); the lateral knee joint X-ray post the modified method surgery (**D**).

In the group of the modified method, the proximal ends of the K-wires were bent toward the patellar by 180°, and we drilled holes in the superior extreme of the patellar with a 2.5-mm K-wire near the corresponding site of the bended part of the 2.0-mm K-wires. Then, we knocked the 2.0-mm K-wires downward to insert the bended part into the holes to prevent the rotation of the K-wires. The specific case surgical techniques are indicated in Figure 1. Preoperative and postoperative radiographs of the specific cases are shown in Figure 2.

**Figure 3:**
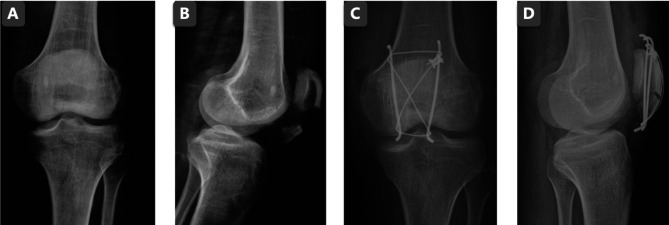
The AP knee joint X-ray before the surgery (**A**); the lateral knee joint X-ray before the surgery (**B**); the AP knee joint X-ray post the traditional method surgery (**C**); the lateral knee joint X-ray post the traditional method surgery (**D**).

In the group of the traditional method, the proximal ends of the K-wires were bent toward the patellar. Preoperative and postoperative radiographs of the specific cases are shown in Figure 3.

### Statistical Method

Summary statistics were presented as frequencies and proportions for categorical variables and means with standard deviations or medians with interquartile ranges for continuous variables depending on data distribution. We compared baseline characteristics between study groups using Student’s *t*-tests or the Mann–Whitney *U* test for continuous variable and *χ*^2^ tests for categorical variables, as appropriate. A score of *p *< 0.05 was considered as a significant statistical difference.

## Results

The traditional tension band method was used in 46 patients, and the modified method was used in 29 patients. The age, gender, and fracture type were comparable (Table 1). With the accumulation of sample size, more detailed baseline data of patient can be further analyzed and compared (9). The data of operation and outcome is presented in Table 2. The operation time and blood loss in the two groups were similar and did not have significant statistical differences (*p *> 0.05). The healing times of the patellar fractures in the two groups were similar (3.9 ± 1.2 months vs. 3.8 ± 1.1 months, *p *= 0.153). The KSS clinical scores 1 year after operation in the traditional method group and the modified method group were 90 (84, 95) and 99 (97, 100), respectively, with a significant statistical difference (*p *< 0.05). The KSS functional scores 1 year after operation in both groups were 90 (65, 90) and 100 (90, 100) with a significant statistical difference (*p *< 0.05).

**Table 1:** Demographic and clinical characteristics of patients of patellar fracture.

	Traditional group (*N* = 46)	Modified group (*N* = 29)	*p-*value
Age (IQR, year)	64 (54, 75)	63 (60, 67)	0.632
Sex [no. (%)]			0.535
Men	24 (52.2)	13 (44.8)	
Women	22 (47.8)	16 (55.2)	
AO classification [no. (%)]			0.554
C1	9 (19.6)	7 (24.1)	
C2	9 (19.6)	3 (10.3)	
C3	28 (60.8)	19 (65.6)	

*IQR, Interquartile range.*

**Table 2:** The data of operation and outcome.

	Traditional group (*N *= 46)	Modified group (*N *= 29)	*p-*value
Operation duration (*μ* ± *σ*, min)	67.6 ± 9.8	63.6 ± 15.4	0.170
Blood loss (*μ* ± *σ*, ml)	93.7 ± 18.0	100.3 ± 27.1	0.205
Healing time (*μ* ± *σ*, month)	3.9 ± 1.2	3.8 ± 1.1	0.805
Complication [no. (%)]	35 (76.1)	2 (6.9)	<0.001
KSS score (clinic, IQR)	90 (84, 95)	99 (97, 100)	<0.001
KSS score (function, IQR)	90 (65, 90)	100 (90, 100)	<0.001

*KSS, knee society score; IQR, Interquartile range.*

The incidences of complications due to the rotation of K-wires in the traditional group and the modified group were 76.1% (35 of 46) and 6.9% (2 of 29), respectively, with a significant statistical difference (*p *< 0.05). We chose the rotation of K-wires as our primary outcome. The subitems of the complications were also analyzed (Table 3).

**Table 3:** The primary outcome and secondary outcome.

	Traditional group (*N* = 46)	Modified group (*N *= 29)	*p-*value
Complication [no. (%)]	35 (76.1)	2 (6.9)	<0.001
Primary outcome			
Rotation of K-wires [no. (%)]	35 (76.1)	0 (0)	<0.001
Secondary outcome			
Simple rotation [no. (%)]	14 (30.4)	0 (0)	0.001
Cable unhooking [no. (%)]	18 (39.1)	1 (3.4)	0.001
Fixation breakage [no. (%)]	1 (2.2)	1 (3.4)	1.000
Skin ulceration [no. (%)]	4 (8.7)	0 (0)	0.269
Skin irritability [no. (%)]	24 (52.2)	0 (0)	<0.001

### Primary Outcome

The average time of onset of primary outcome complications in the traditional method was 3.2 months, and there was no primary outcome complications in the modified method. A total of 35 of the 46 patients in the traditional group suffered the rotation of K-wires, but no patient in the modified group suffered K-wire rotation.

### Secondary Outcome

**Figure 4:**
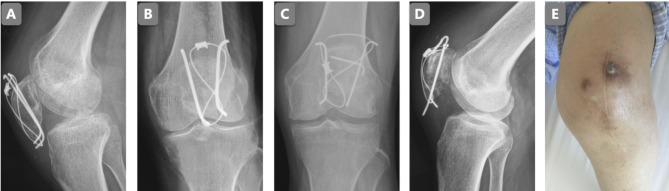
Complications: simple rotation (**A**); cable unhooking (**B**); cable breakage (**C**); K-wire breakage (**D**); skin ulceration (**E**).

Of the 46 patients in the traditional method, 14 patients sustained simple rotation injuries (Figure 4A). There were 18 patients in the traditional method group and 1 patient in the modified method group who sustained cable unhooking injuries (Figure 4B). One patient in the traditional method group and one patient in the modified method group suffered fixation breakage. However, the broken fixation of the two groups was different. Cable breakage (Figure 4C) occurred in the traditional group, and K-wire breakage (Figure 4D) occurred in the modified group. Of the 46 patients in the traditional method group, 4 patients suffered skin ulceration (Figure 4E).

## Discussion

The results of the present study showed that our new method presented excellent antirotation performance, better functional outcomes, and a lower complication rate compared with the traditional technique.

Many operation techniques are applied in clinical practice. Patellar plate, cable pin, headless screw, headed screw, and K-wire tension band are commonly adopted worldwide. Patellar plates have many shapes such as star plate, shield plate, and bilateral fixed-angle plate (10–12). Several studies have demonstrated efficacy in the treatment of plate fractures (13, 14), but most biomechanical studies about patellar plates have used the transverse patellar fracture models. Only a few studies in the literature have focused on the application of the patellar plate to comminuted patellar fractures. Thelen et al. performed cadaver testing of a fixed-angle plate in a comminuted plate fracture (13). Wurm et al. used an angular stable plate to treat a comminuted patellar fracture and achieved an overall complication rate of about 6% (14). But it is difficult to fix screws to comminuted fractures, because the patellar may be too comminuted to have enough space for the screws to hold. The result of the layered patellar fracture using a locking plate was also not satisfactory.

Another category of internal fixator is the tension band. Some of the cables used in the clinic include titanium cable and stainless-steel cable. K-wires, lag screws, cannulated screws, and headless screws are used, along with cables, to enhance strength. Some biomechanical tests have demonstrated the reliability of tension band wiring with screws in transverse patellar fractures (15, 16). But an adverse conclusion was reported in Wagner’s research. The strength of cannulated lag screws with tension band wiring was not found suitable for the biomechanical test and showed a displacement of 2.8 mm after 100 cycles, and this meant implant failure (10). Also, in comminuted patellar fractures, there may not be enough space for the screws to hold. These screws may cause excessive bone loss and additional risk of fracture and make it difficult to fix a small patellar (17, 18). So, screws cannot be used in all types of patellar fractures. In contrast, K-wires have been found to be superior to screws at this point of time. We can even use 1.4-mm K-wires to fix small fragments. Huang et al. supposed that K-wires are essential in the treatment of comminuted patellar fracture, because these wires have the ability to turn comminuted fractures into simple fractures, and they concluded that if there is no K-wire fixation, displacement can easily occur (19). The sturdiness of the tension band with K-wires was testified in a mass of articles and clinical practices (20, 21). With increased movement of the knee in some patients, K-wires will rotate in these patients. In our study, most patients suffered the rotation of K-wires in the first 3 months after operation. From 3 weeks to 3 months after operation, there was swelling of the knee, and it increased over time. Some elderly patients delayed the inevitable by opting for rehabilitation later than 3 months after operation. The rotation of K-wires may lead to a stimulation of the skins of the front knee and also ulceration up to 40% of patients (22). Our data showed that in the traditional method group, 52.2% (24 of 46) patients suffered skin irritability and 8.7% (4 of 46) patients suffered skin ulceration. These complications may hinder rehabilitation. But we found that not all K-wire-rotation patients experienced hardware stimulation. This may be due to the fact that not all rotated K-wires were bent too much or the subcutaneous tissue in front of the patellar in some patients was thicker. To prevent the rotation of the K-wires, we bent the proximal part of the wires to 180° and knocked the bent part into the upper extreme of the patellar. This will enable the fixing of K-wires with the patellar and preventing their rotation. To achieve this objective, we drilled a hole at the point where the bent part will be knocked in at the superior extreme of the patellar with 2.5-mm K-wires after the 2.0-mm K-wires fixed the main fragments. To avoid the titanium cable or stainless-steel cable slipping from the K-wires, we should keep enough K-wires out of the patellar. But the outside part should not be too long to stimulate the skin. In our experience, the appropriate length of the outside part of the K-wires should be 5 mm. The inside part of the K-wires knocked into the patellar should not be too short, else the loop will be overloaded and will crack. Sufficient inside part length can also increase the fixation stability. At least 5-mm wires are recommended on the basis of our experience.

According to Table 2, the average operating time of the modified method was similar to that of the traditional method. Compared with the traditional method, we added the procedures of drilling two holes in the upper extreme of the patellar, knocking the K-wires into the holes and bending the wires to 180°. Usually, these procedures might consume some time, but in our case, the time taken was not long (usually 1–3 min). The amount of blood loss in the modified method group was a little more than in the traditional method, but no statistical difference was found between the two groups. To drill the two holes, a 0.5- to 1-cm long incision had to be made, and this left open the possibility of causing more blood loss during operation. However, the core data of operation between the two groups were similar, so the safety of the modified method was in no way less than that of the traditional method.

Some scholars supposed that the K-wire tension band technique cannot fix comminuted patellar fractures because K-wires do not have sufficient thickness (11), but in our study, we did not find any knee displacement using the modified method during the follow-up, especially in C3-type patellar fractures. Our study result revealed that the KSS score 1 year after surgery in the modified method group was significantly higher than that in the traditional method group. Due to the bending of the K-wires and their embedding into the patellar, their rotation was avoided. To avoid irritation from K-wires, the bent part of the wires should not be left outside of the patellar too much, but if left too short, the cable might unhook from the K-wires. Because we adopted this modified procedure, we did not see any rotation of the K-wires in patients who belonged to the modified method group. So, when these patients underwent rehabilitation and were put in an exercise regimen after the operation, they did not experience any skin irritation symptoms due to the rotation of the K-wires. No limits were imposed during their rehabilitation, and the more the rehabilitation, the higher was the KSS score. But in long-term observation, patients in the traditional group cannot enjoy the advantages offered by the modified method, especially after they remove the internal fixator placed on them.

One patient in the modified method group suffered K-wire breakage, and one patient in the traditional method group suffered titanium cable breakage. In the former group, we found that the breakage was associated with the 180° bending of the K-wires. The K-wires we used were made of titanium. When K-wires made of titanium are bent to a such high degree, the mechanical strength would decline sharply. In the process of flexion–extension, the K-wires might break.

## Conclusion

Based on the present results, we can state that our modified method could effectively avoid the rotation of K-wires, provide improved knee function at 12 months after surgery, and reduce complications resulting from such rotation. Our modified method is easy to perform and may provide a better clinical choice in the treatment of patellar fractures. However, the issue of K-wire breakage due to buckling needs to be addressed. Meanwhile, a prospective randomized controlled trial with a larger sample size is needed to further confirm our results.

## Data Availability

The raw data supporting the conclusions of this article will be made available by the authors, without undue reservation.
